# An Optimal Cure Process to Minimize Residual Void and Optical Birefringence for a LED Silicone Encapsulant

**DOI:** 10.3390/ma7064088

**Published:** 2014-05-27

**Authors:** Min-Jae Song, Kwon-Hee Kim, Gil-Sang Yoon, Hyung-Pil Park, Heung-Kyu Kim

**Affiliations:** 1 Molds & Dies Technology R&DB Group, Korea Institute of Industrial Technology, 7-47, Songdo-dong, Yeonsu-gu, Inchoen 406-840, Korea; E-Mails: mjsong@kitech.re.kr (M.-J.S.); seviaygs@kitech.re.kr (G.-S.Y.); php76@kitech.re.kr (H.-P.P.); 2 Department of Mechanical Engineering, Korea University, Anam-dong, Seongbuk-gu, Seoul 136-713, Korea; E-Mail: kwonhkim@korea.ac.kr; 3 Department of Automotive Engineering, Kookmin University, 77, Jeongneung-ro, Seongbuk-gu, Seoul 136-702, Korea

**Keywords:** LED, silicone resin, encapsulant, multi-step cure process, voids, birefringence, residual stress, cure kinetics

## Abstract

Silicone resin has recently attracted great attention as a high-power Light Emitting Diode (LED) encapsulant material due to its good thermal stability and optical properties. In general, the abrupt curing reaction of the silicone resin for the LED encapsulant during the curing process induces reduction in the mechanical and optical properties of the LED product due to the generation of residual void and moisture, birefringence, and residual stress in the final formation. In order to prevent such an abrupt curing reaction, the reduction of residual void and birefringence of the silicone resin was observed through experimentation by introducing the multi-step cure processes, while the residual stress was calculated by conducting finite element analysis that coupled the heat of cure reaction and cure shrinkage. The results of experiment and analysis showed that it was during the three-step curing process that the residual void, birefringence, and residual stress reduced the most in similar tendency. Through such experimentation and finite element analysis, the study was able to confirm that the optimization of the LED encapsulant packaging process was possible.

## 1. Introduction

Recently Light Emitting Diode (LED) has attracted great attention as a next-generation light source due to its excellent characteristics such as high-energy efficiency, quick response, long life, and light weight. To protect the LED module, transparent thermoset polymers, such as epoxy or silicone resin, are used as encapsulant covering the LED module. Silicone resin is regarded as superior to epoxy in terms of thermal and mechanical characteristics especially for high-power LED. 

Silicone resin is formed into an encapsulant by the consecutive cure process, which involves the mixing of base and curing agent, the casting into a mold, the heat-assisted chemical curing at elevated temperatures, and the post-cure cooling. During the chemical curing, abrupt temperature rise can cause various defects in the cured silicone resin; *i.e*., surface roughening, delamination from LED chip, residual void including air and water, and residual stress, which lead to the deterioration of mechanical and optical properties of LED lighting products. In order to minimize such defects, recently a new cure process of step-by-step temperature rise has been investigated for thermoset polymers. For example, Zhang *et al.* [1] suggested an optimum cure condition for semiconductor encapsulant epoxy resin by applying a step cure condition and three-dimensional finite element analysis. 

White and Hahn [2,3] proposed an optimal cure cycle that could reduce residual stress during the cure of composite structures. Kim and Hahn [4] investigated the residual stress build-up by the intermittent curing of the unsymmetrical graphite–epoxy laminates using the elasticity-based approach to relate the experimental warpage to the residual stresses. Bogetti and Gillespie [5] analyzed the development of residual stresses in the cured thick composite laminates by coupling the incremental laminated plate theory with the one-dimensional cure simulation, accounting for the chemical shrinkage based on the cure-dependent mechanical property of thermoset resin. Zhu *et al.* [6] presented a full three-dimensional coupled thermo-chemo-viscoelastic model simulation for the heat transfer, curing, residual stresses, and deformation in a composite part. Yi *et al.* [7] developed a nonlinear transient heat transfer FE model, where material parameters were both temperature and Degree of Cure (DOC) dependent, and applied the FE analysis to the two-dimensional cure simulation. 

Ciriscioli *et al.* [8] developed an algorithm to minimize the internal residual voids and stress for a composite. In the multi-step cure process, the abrupt temperature rise due to chemical cure reaction heat in the cured polymer can be avoided since the initial temperature for chemical curing is lower than the final temperature of the cure process. As a result, various defects in the encapsulant due to abrupt temperature rise can be considerably reduced by virtue of the gradual temperature rise.

When the step cure process was applied in the previous study, how the residual voids decreased was observed, through which it was understood that the step cure process was effective for at least decreasing the residual void [9,10]. In this paper, whether the step cure process was also effective in terms of reducing the residual stress and birefringence was quantitatively studied by calculating the residual stress through the finite element analysis of the cure process and experimentally measuring the birefringence. In addition, the optimal cure process was derived by conducting an optimization analysis. 

## 2. Experimental Observation

### 2.1. Experiment Apparatus 

**Figure 1:**
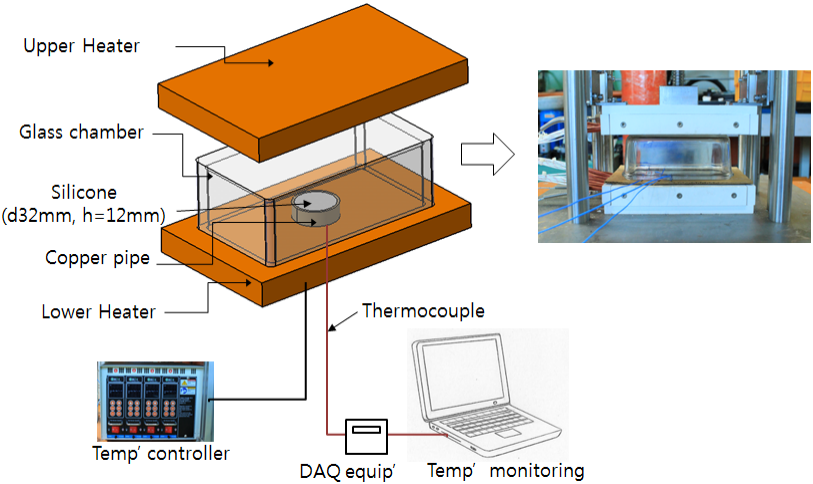
Experimental set-up for silicone molding.

Figure 1 shows the experimental set-up for cylindrical silicone molding. For the convenience of experiment and analysis, cylindrical silicone resin with axial symmetry, diameter of 32 mm, and height of 12 mm was used. The experiment apparatus consisted of lower heating plate, upper heating plate, temperature controller, thermocouple, and real-time temperature monitoring device. In addition, the accuracy of the test was increased by adding a glass cover to the molding shape to prevent heat loss due to the outside air. The molding was mainly done with the lower heating plate while the upper heating plate was heated and kept at 30 °C to minimize any error due to the heat transfer with the outside air. The cure process was comprised of mixing liquid silicone base and curing agent, casting into a mold, cure reaction at specific temperature, and room temperature cooling. In order to clearly observe the residual voids, the base and the curing agent were agitated and directly cast without undergoing the vacuum degassing process. 

### 2.2. Step Cure Process Design 

**Figure 2:**
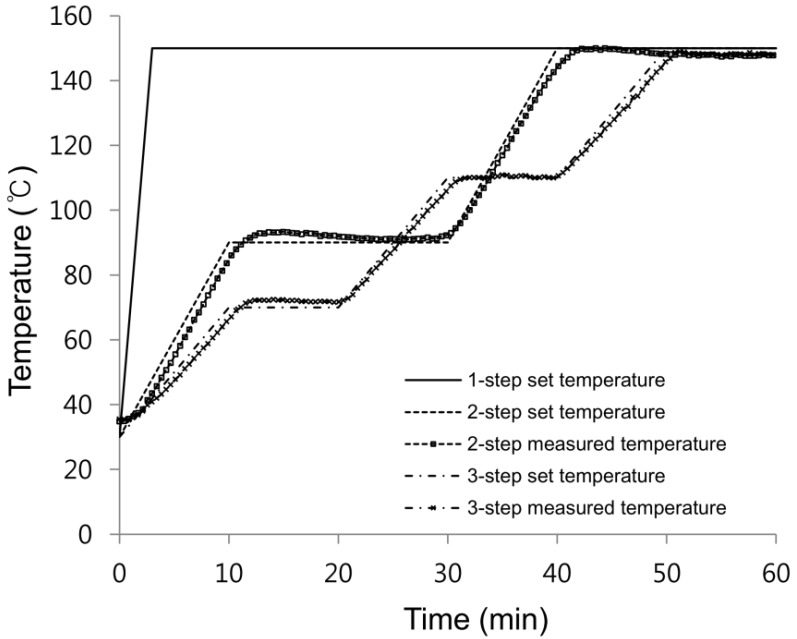
Temperature-time cure cycle ramps.

In order to determine the curing temperature and step cure conditions, the cure reaction temperature of the heating-up reaction was measured by making use of differential scanning calorimetry (DSC). The silicone resin generated reaction heat due to the chemical reaction during the curing process into thermoset resin and when this generated reaction heat was measured with DSC, the curing properties could be made known [11]. In the case of the silicone used in this study, the cure reaction began at 70 °C and finished at 150 °C [9,10]. The curing conditions as shown in Figure 2 and the actual temperature measuring profile were derived by making reference to the cure reaction results. Although there were slight differences with the conceptualized curing conditions, it was observed that the temperature distribution was relatively well-followed. A total of three types of curing conditions were applied wherein curing was done at an isothermic condition of 150 °C in the one-step, there was temperature elevation to 90 °C and application of curing time in the two-step, and there was temperature elevation to 70 °C and 110 °C and application of curing time in the three-step. 

### 2.3. Residual Voids 

Among the defects in LED products, the residual voids of the curing process were a cause of decreasing the LED lamp efficiency. Such residual voids typically occur when the air is mixed during the dispensing or the silicone is abruptly cured due to the mixing of moisture and vaporization of the moisture in the adherend during the silicone storing process [9,10]. After conducting the step cure experiment according to Figure 2, microscopic imaging at a magnification of 40× was performed on the cured specimen to observe the presence of residual void. 

**Figure 3:**
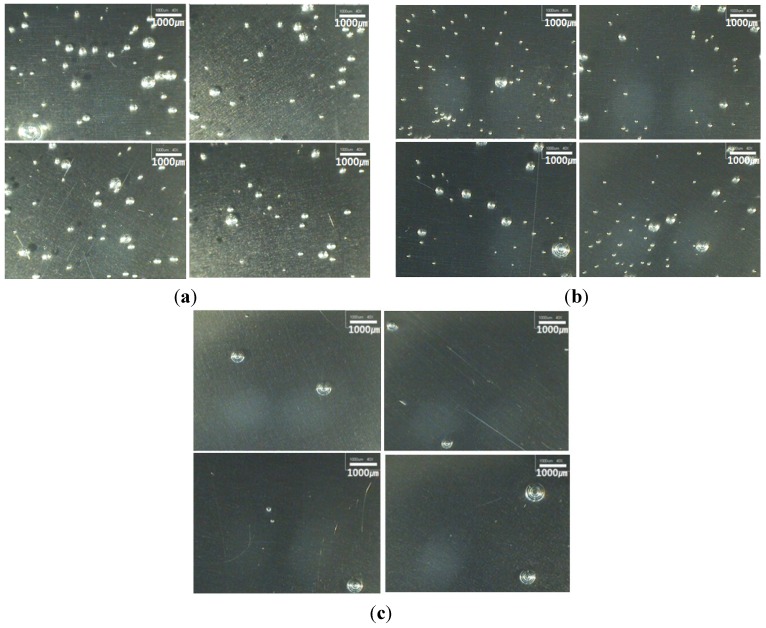
Trapped voids after step cure process. (**a**) one-step; (**b**) two-step; (**c**) three-step.

Figure 3 shows the residual voids’s distribution from the step cure process. Figure 3a shows the distribution in the one-step, which refers to the high-temperature isothermal cure process at 150 °C. As shown in the figure, voids that were pre-existing in the material, voids that formed during the casting process, and the many voids that formed as moisture in the adherend, vaporized as abrupt cure reaction began. Figure 3b shows the results of the second step cure. In comparison to the first step cure, relatively large voids were removed but it was observed that small voids still remained. Figure 3c shows the results of the three-step cure. Although some of the large voids remained, it was found that most of the voids were removed during the 70 °C heating-up stage and isothermal stage, which were approximately 20 min. It is considered that internal voids are sufficiently removed when applying the step cure process from low temperature to high temperature since the viscosity is maintained at low enough state prior to curing, after which curing with almost no voids present would be possible during the reaction. 

## 3. Finite Element Analysis of Silicone Curing for Step Cure Process Analysis

### 3.1. Governing Equation and Cure Kinetics 

The finite element analysis was conducted to predict the degree of cure and temperature distribution according to the step cure conditions. The three-dimensional heat transfer energy equation is the sum of the Fourier heat conduction equation and the exothermic reaction from the cure reaction, which can, in summary, be expressed as follows [1,12]:

(1) $$\text{ρ}C_{\text{p}}\frac{\partial T}{\partial t}=k\left(\frac{\partial^{2}T}{\partial x^{2}}+\frac{\partial^{2}T}{\partial y^{2}}+\frac{\partial^{2}T}{\partial z^{2}}\right)+\text{ρ}H_{\text{r}}\frac{\text{dα}}{\text{d}t}$$

Here, *T* stands for absolute temperature, *k* for thermal conductivity, *C*_p_ for specific heat, ρ for density, *H*_r_ for total heat of reaction, α for degree of cure, and *t* for time. In order to find the reaction rate, the isothermic cure experiment using the Differential Scanning Calorimeter (DSC) was performed at temperatures of 60 °C, 70 °C, 80 °C, and 90 °C. Based on such test results, the constants of the autocatalyzed cure kinetics were obtained as shown in Equation (2) [13]:

(2) $$\frac{\text{dα}}{\text{d}t}=K{\text{α}}^{m}{\left(1-\text{α}\right)}^{n}$$

(3) $$K=A\exp\left(-\frac{E}{RT}\right)$$

Here, α stands for degree of cure, *K* for reaction rate constant, *m* and *n* for reaction exponent, *A* for pre-exponential factor, *E* for active energy, *R* for gas constant, and *T* for absolute temperature. Table 1 shows the constants of the cure kinetics that were obtained by conducting non-linear curve fitting [10,11].

**Table 1:** Constant of cure kinetics for silicone.

Constant	Cure temperature (°C)			
	60	70	80	90
*k* (×10^−3^)	1.736	3.839	9.739	21.58
*A* (S^−1^)	4.007 × 10^10^			
*E* (KJ/mol)	85.293			
*m*	0.62			
*n*	1.39			
R (J/mol·K)	8.31			

### 3.2. Assumption of Elastic Modulus and Cure Shrinkage 

**Figure 4:**
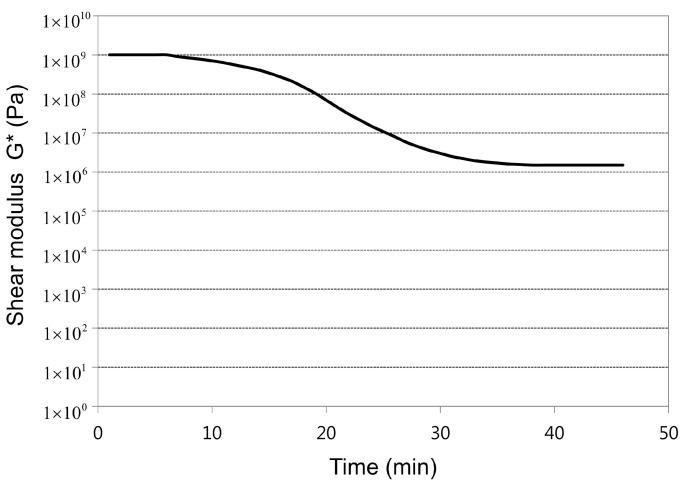
Stress relaxation of silicone resin.

Silicone is a visco-elastic material wherein the modulus changes according to time and temperature. Accordingly, Figure 4 shows the stress relaxation curve of the storage modulus which was obtained by experimenting with dynamic mechanical analysis (DMA). Although the curve fitting must be done with Prony series as shown in Equation (4) within the linear visco-elastic region for the analysis, only the effects of elasticity were considered since silicone was a material in which the cure process took place from liquid state to solid state and the effects of visco-elasticity in the cure process was not significant [2,3].

(4) $$G\left(t\right)=\frac{\text{σ}\left(t\right)}{{\text{ε}}_{0}}=G_{0}+\sum_{i=1}^{n}G_{i}\exp\left(-\frac{t}{{\text{τ}}_{i}}\right)$$

Here, σ stands for stress, ε for strain, *t* for time, *G*_0_ for equilibrium modulus, *G_i_* for *i-*th Prony constant, and τ*_i_* for *i-*th Prony retardation time constant. 

The silicone resin is gradually cured from liquid phase to solid phase and its elastic modulus increases accordingly to which Tsygankov *et al.* [2,3,14] reported that there was linear relationship between the degree of cure and the elastic modulus. Therefore, the relationship between the degree of cure and the elastic modulus E can be expressed as shown in Equation (5):

(5) $$E=E_{0}\text{α}$$

Here, *E*_0_ stands for the proportional constant. 

In case of thermoset resin, cure shrinkage occurs due to the chemical crosslink reaction according to the progress of cure because of which volumetric reduction takes place [15]. In general, it is known that the cure shrinkage has linear relationship with degree of cure [5,16] and so modeling the degree of cure with linear function is possible as shown in Equation (6):

(6) $$V=\text{α}V^{T}$$

Here, *V* stands for volumetric cure shrinkage, α for degree of cure, and *V^T^* for total volumetric cure shrinkage. It is known that the silicone used in this study has cure shrinkage of approximately 3% upon complete cure.

### 3.3. Thermal Properties 

The thermal properties were obtained as shown in Table 2 to conduct the heat transfer analysis. The measurements were done by measuring three times using laser flash after which the average value was taken.

**Table 2:** Thermal properties of silicone.

Thermal properties	Value
Density (kg/m^3^)	1168
Thermal conductivity (W/m·°C^−^^1^)	0.148
Specific heat (J/kg·°C^−^^1^)	1424

In addition, for the initial condition, room temperature (30 °C) was applied for all joints, and for the boundary condition, natural convection condition was assumed in which the silicone resin was heated to set temperature by the lower heating plate while the upper plate was not heated additionally. The heat transfer coefficient between the silicone and the cylindrical heater during the heating up process was assumed to be 0.5 KW/m^2^·°C. The stress-strain that occurred in the silicone resin during the cure process was calculated by coupling the structural analysis, which considered both the elastic deformation and cure shrinkage, with the heat transfer analysis that considered the heat of cure reaction. Furthermore, the measurements were taken from the central part of the cylindrical model and the wall side of the mold for comparing the curing characteristics according to position.

## 4. Analysis and Experiment Results on the Step Cure Process

### 4.1. Finite Element Analysis for Step Cure 

**Figure 5:**
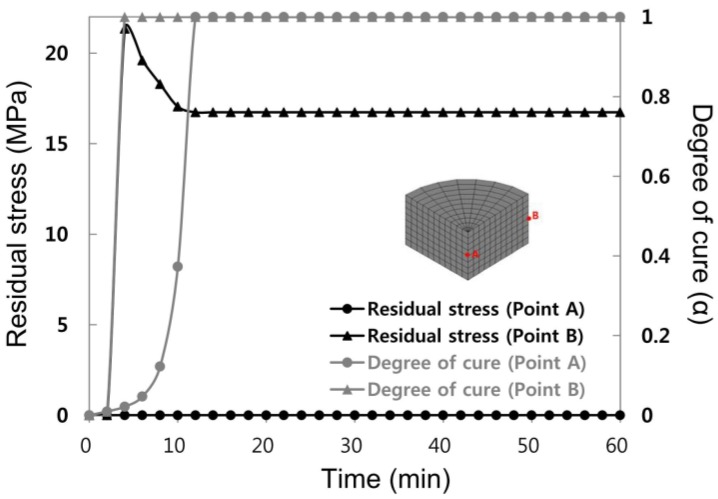
Residual Stress evolution and degree of cure during one-step curing at Points A and B.

Figure 5 illustrates the results of residual stress and degree of cure distribution for one-step curing. The analysis results showed that residual stress largely appeared at the wall side of the mold, but it was not observed from the central area of the mold. The residual stress at the wall side of the mold (point B) increased to a maximum after four minutes, followed by decreases, before it maintained a constant value after 12 min. The degree of cure for four minutes elapsing at these two sides showed that the wall side was completely solidified due to abrupt curing process on four minutes of curing time, while curing progressed in the central area (point A) of the mold. Therefore, there occurred a large difference in the degree of cure according to location, which led to the solidified condition. It was found that large residual stress occurred due to cure shrinkage from the solidified wall side of the mold. After 12 min, curing was completed until the central area. Cure shrinkage with complete solidification until the central area might have reduced the residual stress, which was maintained at the wall side of the mold to some extent. 

**Figure 6:**
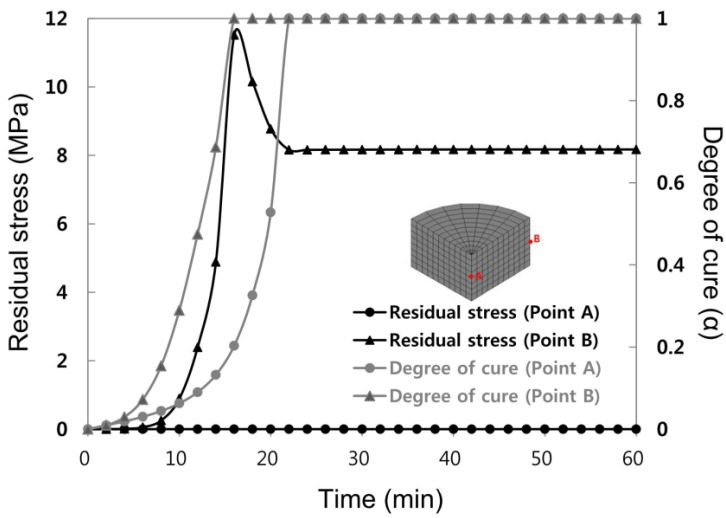
Residual Stress evolution and degree of cure during the two-step curing at Points A and B.

Figure 6 shows the results of residual stress and degree of cure distribution for two-step curing. The analysis results showed that residual stress largely appeared as in one-step, while residual stress was not observed from the central area (point A) of the mold. When the degree of cure at two areas was compared during 16 min of curing time, 100% solidification was achieved from the wall side (point B) due to abrupt cure reaction showing complete solidification, while the central area was solidified only around 20%. The difference in the degree of cure according to location the same as one-step resulted to different solidification conditions in different locations. Larger residual stress was observed from the solidified wall side of the mold. After 22 min, solidification was completed until the central area, while curing process was completed which might reduce residual stress and maintain it to some extent.

The final residual stress value for Point B (wall side) was 11.5 MPa, which reduced from the first step analysis result of 21.3 MPa by 54%. The final stress value was 8.2 MPa, which reduced from the first step analysis result of 8.2 MPa by 49%.

**Figure 7:**
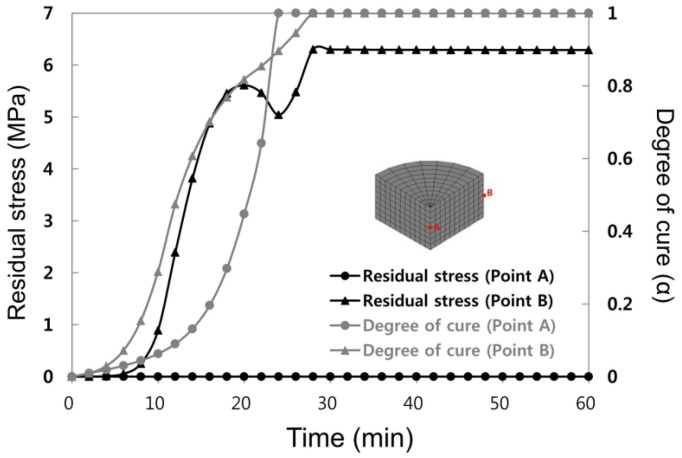
Residual stress evolution and degree of cure during the third step curing at Points A and B.

Figure 7 illustrates the results of residual stress and degree of cure distribution for three-step curing. The analysis results showed that residual stress was observed largely from the wall side of the mold, holding the same results as earlier, while residual stress was not found from the central area (point A) of the mold. However, unlike in the previous case, residual stress during the initial stage of curing was not abruptly increased in the wall side (point B) of the mold. Solidification was completed in the central area of the mold for four minutes more than in the wall side when the degree of cure progressed from 90% due to gradual temperature increases. Therefore, it can be judged that abrupt increase in residual stress was mitigated since the degree of solidification according to location became minimized as compared with earlier solidification progress. The final residual stress value at point B (wall side) was 6.29 MPa, which decreased from the one-step analysis result of 21.3 MPa by 38%. 

**Figure 8:**
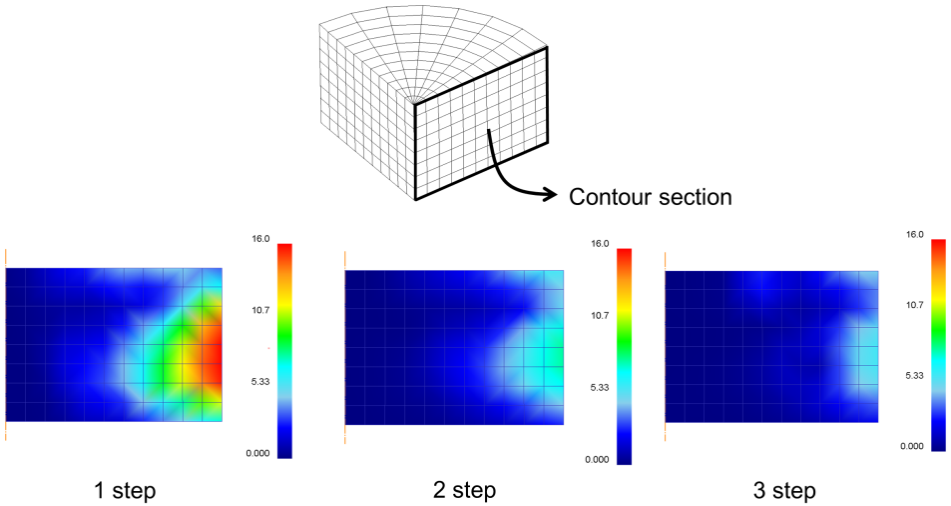
Section contour of residual stress in step cure process.

Figure 8 shows the section contour of the final residual stress distribution. As shown in the figure, there was almost no stress generated in the central area but there was high stress generated in the wall side of the mold. In addition, it was evident that as the number of steps increased, the final residual stress greatly decreased. 

Table 3 shows the final residual stress occurring during each step. During one-step; that is, during isothermal cure, relatively large residual stress occurred near the wall side since the degree of cure was different at different locations due to abrupt temperature increases and exothermic reaction generated inside of the mold, while cure reactions during two-step and three-step yielded relatively small residual stresses and the difference per the location was also less. The abrupt cure reaction would not occur under these cure condition steps because temperature was not abruptly increased.

**Table 3:** Comparison of final residual stress.

Measuring point	Step cure	Final Residual Stress	Difference
Point A	1 step	0 MPa	No residual stress generated in the center
	2 step	0 MPa	
	3 step	0 MPa	
Point B	1 step	16.73 MPa	–
	2 step	8.17 MPa	49% of 1-step
	3 step	6.29 MPa	38% of 1-step

### 4.2. Results Comparison for Birefringence Distribution and Analysis 

A residual stress in transparent products such as lenses causes not only deterioration of optical properties but also increases in optical distortion [17,18]. Lu and Khim [19] carried out injection molding experiment using the design of experiment for polycarbonate spherical lenses. They estimated the distribution of residual stress for lenses according to process condition by the measurement of birefringence. They also improved the optical property of lenses by improving the unevenness on the surface which was caused by residual stress. 

Lai and Wang [20] predicted residual stress distribution by comparing injection molding analysis and birefringence for COP plastic lenses. They also analyzed the effect of birefringence on the optical property of lenses by measuring the optical aberrations of lenses according to process conditions using design of experiment.

The final residual stress distribution obtained through the analysis can predict and compare the stress distribution through the birefringence test. The linear photo-elastic theory pertains to the phenomenon in which the isotropic material in transmittance optically shows anisotropy while under stress. When material with such characteristics is compressed, characteristics of negative uniaxial crystal are presented whereas characteristics of positive uniaxial crystal are presented in case tensile force is applied instead. For each of these cases, the ray of light corresponds to the direction of stress while the birefringence induced by the anisotropy is proportional to the difference of principal stresses. Therefore, if the distribution of stress for a certain specimen is not consistent, the birefringence will also appear differently. Such photo-elasticity theory provides the foundation for studying the stress distribution of transparent mechanical structures. When the linear photo-elastic theory is expressed as an equation, it is as shown below:

(7) $$n_{i}-n_{j}=C\left({\text{σ}}_{i}-{\text{σ}}_{j}\right)$$

(8) $$\Gamma =t\left(n_{i}-n_{j}\right)$$

*n_i_* − *n_j_* is the birefringence, *t* is the thickness of specimen and Γ is the phase shift (retardation). At a state of plane stress, σ*_i_* and σ*_j_* stand for each of the principal stresses in the two directions, *n_i_* is the refractive index of the light polarized in the main axis *i* direction, and *C* is the stress-optical coefficient. This linear photo-elastic interaction formula has been proven in considerably wide ranges of several polymers [21]. Therefore, phase shift of birefringence as shown in Equation (7) can be a big indicator for predicting the residual stress based on the photo-elasticity theory.

**Figure 9:**
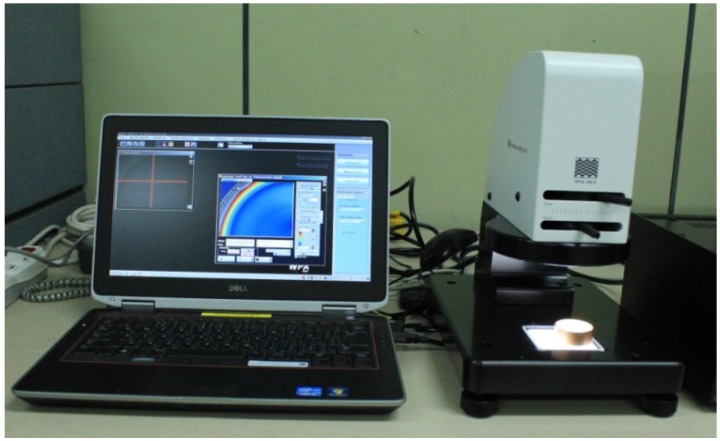
Polarimeter system for measuring residual stresses in silicone encapsulant.

As shown in Figure 9, the phase shift of birefringence of the silicone was measured with WPA-100-S of Photonic Lattice, Inc. (Sendai, Japan). Unlike other existing birefringence measuring devices, this device does not require for the polarized light filter to be rotated. Instead, it carries the advantage of allowing the phase shift of birefringence to be measured quickly and accurately by having an image sensor integrated with fine polarized filter without the polarization drive unit [22]. 

**Figure 10:**
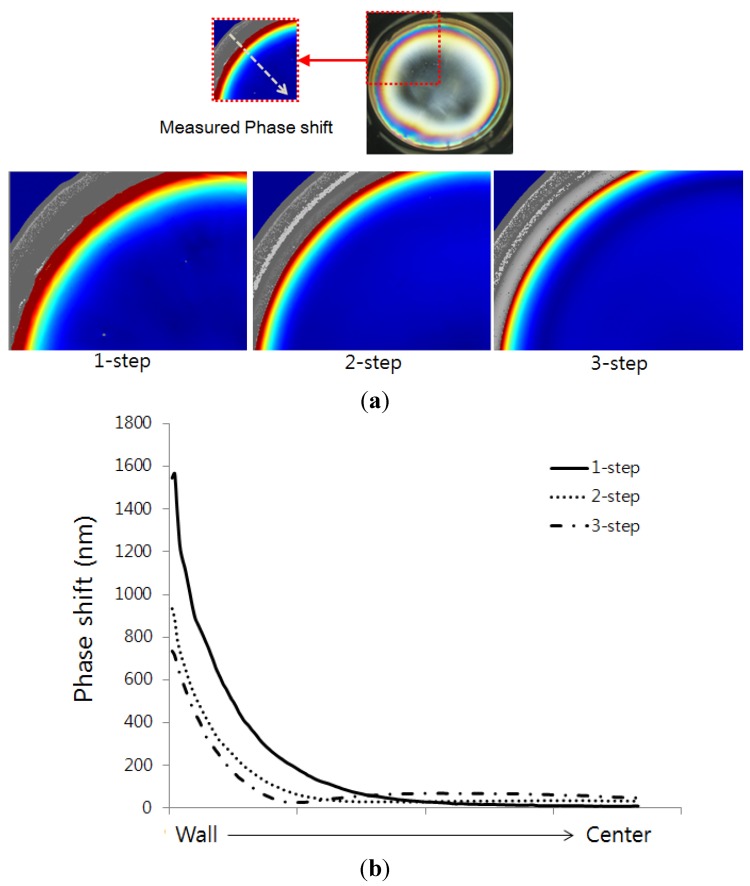
Comparison of birefringence distribution and phase shift after the step cure process. (**a**) Birefringence distribution; (**b**) Phase shift.

Figure 10 shows the birefringence distribution and phase shift according to each step. As illustrated in Figure 10a, the final residual stress is largely distributed from the surroundings of the wall side of mold at first step, and this value decreases more and more as the third step is reached. Figure 10b presents the phase shift value from the wall side of the mold to the center as a graph. There is barely any production of residual stress when moving towards the center and such tendency shows similar distribution with the results from the finite element analysis of the section in Figure 8. 

**Figure 11:**
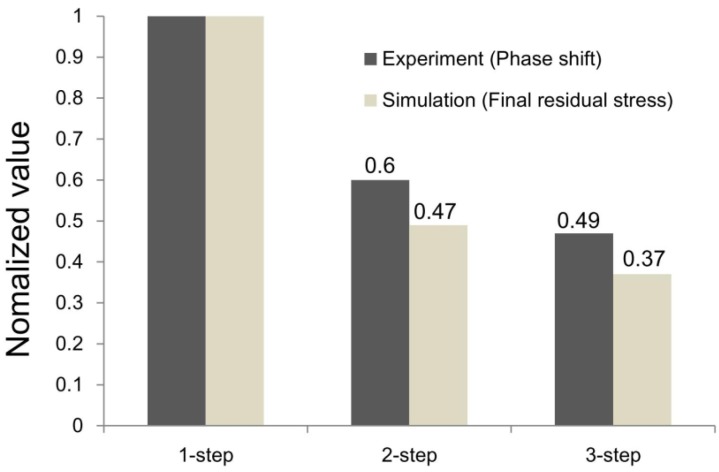
Comparison of normalized value between experiment and simulation.

Figure 11 shows the results of comparing the simulation and experiment as measured from the wall side of the mold. In other words, the final residual stress values based on the maximum value of the first step and the maximum phase shift value of the birefringence were compared non-dimensionally. 

It can be observed that the residual stress and the phase shift decreased similarly as the steps increased. In case of the second step, the residual stress and phase shift accounted for 49% and 59.6%, respectively, in relation to the first step. In case of the third step, the residual stress and phase shift decreased to 38% and 46.9%, respectively, in relation to the first step. Although there were slight differences between the simulated results and experiment results, the reduction of the residual stress with similar tendency was able to be confirmed.

## 5. Three-Step Cure Optimization

### 5.1. Finite Element Analysis for Step Cure 

The Taguchi method was conducted to find the optimal process for understanding the factors that affect the final residual stress in the three-step process and minimizing the final residual stress. 

With the Taguchi method, data is obtained using the table of orthogonal arrays with the controllable design parameters that have significant influence, based on which optimal values are determined by analyzing the SN Signal-to-Noise (SN) ratio. The SN ratio is a scale that considers both the average and scatter of the objective function and is presented as a ratio of the predictive value for the square of population mean for the scatter predictive value. All stages of the Taguchi method aims at reducing the scatter. Reducing the scatter is the same as increasing the SN ratio. Depending on the problem, the SN ratio is defined as nominal-the-best (NTB), smaller-the-better (STB), and larger-the-better (LTB) characteristics. For this study, it can be defined as the smaller-the-better (less residual stress during the cure process is better). The SN ratio is expressed in the following:

(9) $$\text{SN}=-10\log\left(\frac{1}{n}\sum_{i=0}^{n}y_{i}^{2}\right)$$

Here, *n* represents repeated test and *y_i_* represents reaction value.

**Figure 12:**
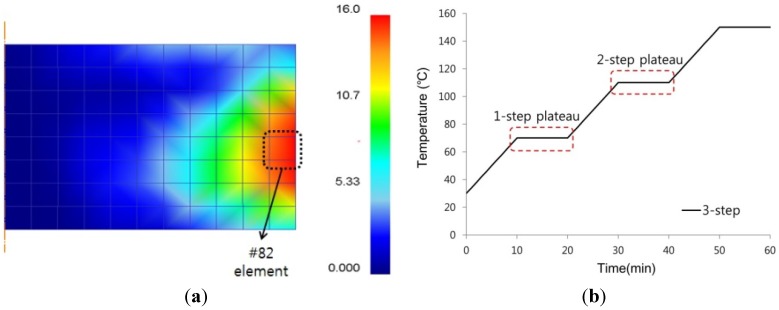
Selection factor for the design of experiment (DOE) of the three-step cure process. (**a**) Measuring element; (**b**) Selection factor.

The target characteristic of the experiment was set as the final residual stress measurement of the #82 element as shown Figure 12a. The heating rate was fixed at 4 °C/min and the factors were selected as shown in Figure 12b wherein the keep temperature and the keep time were selected based on the first step plateau and second step plateau. Table 4 summarizes the selected factors.

**Figure 13:**
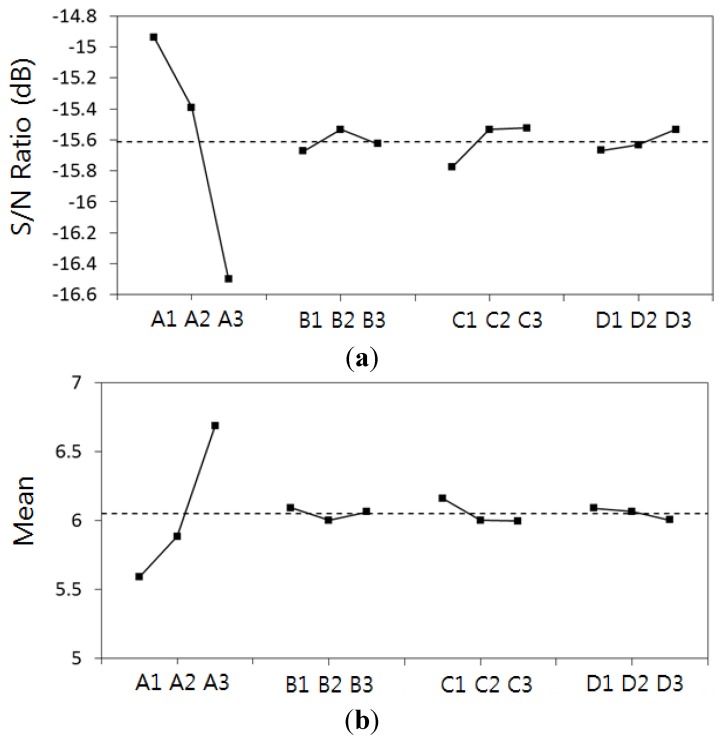
Main effect plots of (**a**) S/N ratio and (**b**) mean value in response graph.

The plan of experiment was prepared using the table of orthogonal arrays of four factors and three levels (L_9_). Table 5 summarizes the results from analyzing each of the experiment conditions of the table of orthogonal arrays. Figure 13 shows the response graph of the S/N ratio and mean value for each of the control factors that were calculated based on the results of the table of orthogonal arrays to find the optimal conditions. The results of the analysis showed that the factor that has the greatest effect on the final residual stress is the first step keep temperature. This result showed that the greatest effect for the final residual stress was during the initial cure temperature, which was similar to the study results in which most of the voids were removed during the initial cure temperature as well [10]. Therefore, it is considered that the residual voids and final residual stress are closely related depending on such process conditions. 

**Table 4:** Design parameters of the DOE of the three-step cure process.

Design parameters		Level		
		1	2	3
A	First step temperature	60 °C	70 °C	80 °C
B	Second step temperature	100 °C	110 °C	120 °C
C	First step time	5 min	10 min	15 min
D	Second step time	5 min	10 min	15 min

**Table 5:** Orthogonal array (L_9_) of the DOE of the three-step cure process.

No.	Design parameters				S/N Ratio (dB)	Final residual stress (MPa)
	First step temperature (°C)	Second step temperature (°C)	First step time (min)	Second step time (min)		
1	60	100	5	5	−15.22	5.77
2	60	110	10	10	−14.81	5.50
3	60	120	15	15	−14.79	5.49
4	70	100	10	15	−15.30	5.82
5	70	110	15	5	−15.28	5.81
6	70	120	5	10	−15.59	6.02
7	80	100	15	10	−16.49	6.68
8	80	110	5	15	−16.51	6.69
9	80	120	10	5	−16.49	6.68

Moreover, the optimal combination for reducing the final residual stress is A1(60 °C)-B2(110 °C)-C3(15 min)-D3(15 min), and this combination was used for conducting optimization analysis. Through the analysis, the final residual stress was calculated to be 5.47 MPa, which was 93.9% of the initial three-step stress value, making it possible to predict an additional 6.1% reduction of final residual stress. 

## 6. Conclusions 

By applying the step cure process of the silicone resin for LED encapsulation, the typical defect of having residual voids and birefringence was observed through experimentation, and the residual stress distribution was calculated by conducting finite element analysis. The results of residual voids experiment showed that most of the voids were removed during the initial cure temperature, which was a temperature elevation to 70 °C and keeping of the isothermic temperature. In addition, the residual stress that occurred in the silicone resin was calculated by coupling the structural analysis that considered the elastic deformation and cure shrinkage with the heat transfer that considered the heat of cure reaction. The results of the analysis showed that the final residual stress decreased by 50% and at most 60% for the two-step and three-step, respectively. Furthermore, the reliability of the analysis was obtained since similar results of birefringence measurement were observed in both the experiment and the simulation. 

In addition, the final residual stress analysis was performed with the Taguchi method by selecting four-process parameters regarding the three-step process. The factor that has the greatest effect on the final residual stress is the first step keep temperature, which is congruent to the similar results of the voids removal experiment. It is considered that such defect factors occur because they are closely inter-related. In addition, it is possible to predict an additional 6.1% reduction in final residual stress through the optimization analysis. 

It is possible to confirm that the optimization of residual stress and optical characteristics of the LED encapsulant is indeed possible through such experiment and finite element analysis. In addition, even in case that the silicone resin is decided to be of specific material, it will be possible to derive the cure process for manufacturing the optimal LED packaging by carrying out the finite element analysis beforehand on the various cure processes (changes in the cure temperature, curing time, cooling time, heating method, *etc.*) based on an understanding of the material properties. 
